# Visual analysis of lung neuroendocrine tumors based on CiteSpace knowledge graph

**DOI:** 10.3389/fendo.2023.1214404

**Published:** 2023-09-06

**Authors:** Mingjie Guo, Shaowen Hu, Yaifei Xiao, Zhan Cao, Zhichao Huang, Yalong Liu, Xiaokang An, Guoyu Zhang, Xianjie Zheng

**Affiliations:** ^1^ Department of Thoracic Surgery, The First Affiliated Hospital of Henan University, Kaifeng, China; ^2^ Department of Clinical Medicine, Medical School of Henan University, Kaifeng, China; ^3^ Department of Neurology, The Fifth Affiliated Hospital of Zhengzhou University, Zhengzhou, China

**Keywords:** lung neuroendocrine tumor, MEN1, ectopic ACTH sign, treatment, CiteSpace

## Abstract

**Objective:**

The relevant literatures in the field of pulmonary neuroendocrine tumor were analyzed to understand the lineage, hot spots and development trends of research in this tumor.

**Method:**

The Web of Science core collection was searched for English-language literature about neuroendocrine tumors of the lung published between 2000 and 2022. CiteSpace software was imported for visualization analysis of countries, institutions, co-cited authors and co-cited journals and sorting of high-frequency keywords, as well as co-cited references and keyword co-occurrence, clustering and bursting display.

**Results:**

A total of 594 publications on neuroendocrine tumours of the lung were available, from 2000 to 2022, with an overall upward trend of annual publications in the literature. Authors or institutions from the United States, Italy, Japan and China were more active in this field, but there was little cooperation among the major countries. Co-cited references and keyword co-occurrence and cluster analysis showed that research on diagnostic instruments, pathogenesis, ectopic ACTH signs, staging and prognosis and treatment was a current research hotspot. The keyword bursts suggested that therapeutic approaches might be a key focus of future research into the field for pulmonary neuroendocrine tumors.

**Conclusion:**

Over these 20 years, research related to neuroendocrine tumors of the lung has increased in fervour, with research on diagnostic instruments, pathogenesis, ectopic ACTH signs, staging and prognosis, and treatment being the main focus of research. Therapeutic treatments may be the future research trend in this field.

## 1 Introduction

Pulmonary neuroendocrine tumours are heterogeneous malignancies arising from endocrine cells and involving different solids, ranging from well-differentiated to highly undifferentiated tumours, including typical carcinoid tumours (TC), atypical carcinoid tumours (AC), high-grade large cell neuroendocrine carcinoma (LCNEC) and small cell carcinoma (SCLC) (1, 2). Of these, TC and AC were classified as high-differentiated low-grade NETs, and LCNEC and SCLC were classified as low-differentiated high-grade NETs. The incidence and prevalence of patients with neuroendocrine tumor of the lung increased annually over the last decades in different countries and regions, while there were differences in survival rates (3–5). In recent years, research on pulmonary neuroendocrine tumors has been increasing, and not only has the classification of the main subtypes been established, but also in-depth studies have been conducted on their pathogenesis, extrapulmonary clinical symptoms and treatment, which provide a solid theoretical basis for mechanism research and treatment tools for pulmonary neuroendocrine tumors.

CiteSpace was a tool frequently employed for bibliometric analysis, providing more than the insights of traditional literature reviews (6). The knowledge map it constructs belongs to documentary scientometrics and is a visual representation of scientific knowledge, capable of mining, analysing and summarising the development process and structure of knowledge in the temporal and spatial dimensions. We aim to use CiteSpace to provide a visual analysis of research trends and hotspots in lung neuroendocrine tumours, with the goal of providing a reference for research in this field.

## 2 Data collection and research methods

### 2.1 Data source and collection

On 19 February 2023, we collected articles in the Web of Science Core Collection (WoSCC) from 2000-01-01 to 2022-12-31 in the field related to neuroendocrine tumors of the lung published in English. Using SCI-EXPANDED, CPCI-S, CPCI-SSH, BKCI-S, BKCI-SSH as data sources, publication types were limited to “articles”. The primary search terms were set to “bronchial neuroendocrine tumor”, “bronchial carcinoid tumor”, “ pulmonary carcinoid”, “pulmonary typical carcinoid”, “pulmonary atypical carcinoid “, “Pulmonary neuroendocrine tumor”, “lung neuroendocrine tumor*”, “neuroendocrine tumor of the lung”. The detailed search strategy was described in the **Supplementary Material (S1)**. The authors, MG and YX independently retrieved the relevant literature from the WoSCC database and then saved and downloaded the searched publications in the “Full Record with Cited References” format. After literature screening, 594 papers were finally obtained as the sample to be visualised and analysed.

### 2.2 Research methods

A Java-based bibliometric analysis visualisation software designed by Professor Chaomei Chen, CiteSpace is an interaction analysis tool to analyse the basic indices of the document that include countries and regions, journals, institutions, keywords and co-citation of references, to build visual graphs for analysis, and to capture keywords and co-cited references with bursts (7). The CiteSpace software version that was used in this study was 6.1.R6 (64-bit). CiteSpace was set up with the following parameters: to perform time slices (1 year per slice) from January 2000 to December 2022, to select all options in the term source, to select one node type at a time, and to select TOP50 as the standard, with the other setting items as default values.

Microsoft Office Excel 2016 (Microsoft, Redmond, Washington, USA) was used to process data and generate statistical charts.

## 3 Result

### 3.1 Quantitative analysis of basic information

#### 3.1.1 Annual growth trends in publications

**Figure 1:**
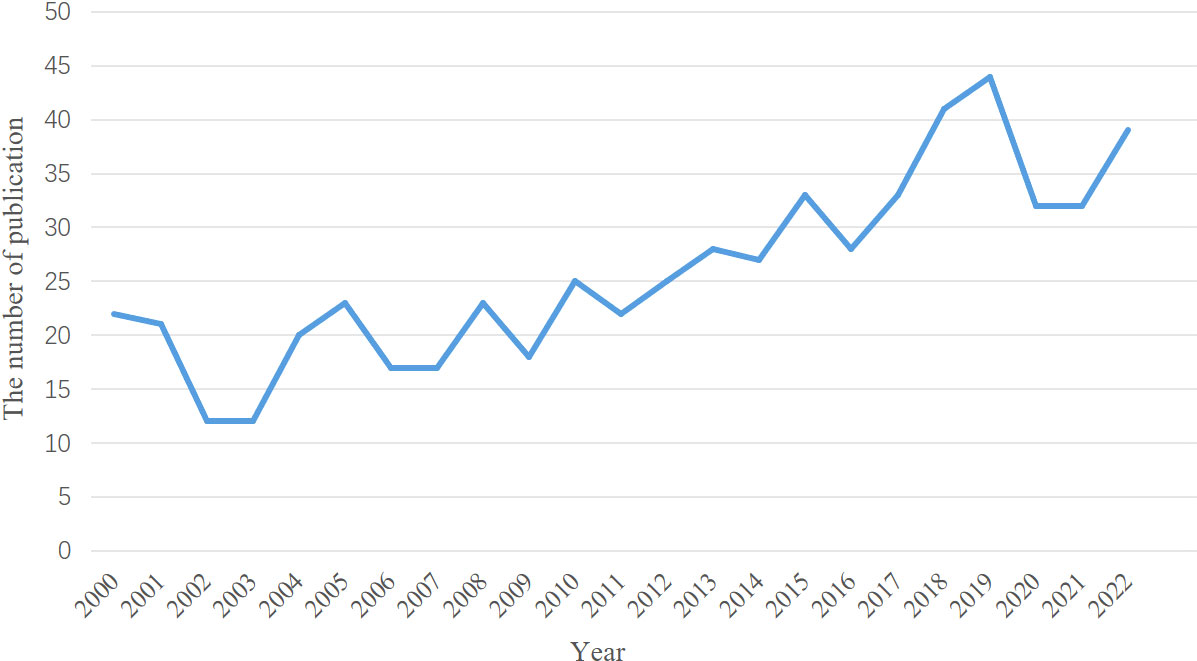
Annual number of publications in Lung Neuroendocrine Tumors.

The WoSCC database search yielded a total of 594 papers on lung neuroendocrine tumour research published between 2000 and 2022. The number of publications declined significantly in certain individual years between 2000 and 2022, as indicated in **Figure 1**, although the overall trend was essentially upward. The year with the fewest papers published was 2002 and 2003 (n = 12, 2.02%), while the year with the most articles published throughout the search period was 2019 (n = 75, 12.63%), with an average of 26 articles published each year.

#### 3.1.2 Country/region analysis

**Figure 2:**
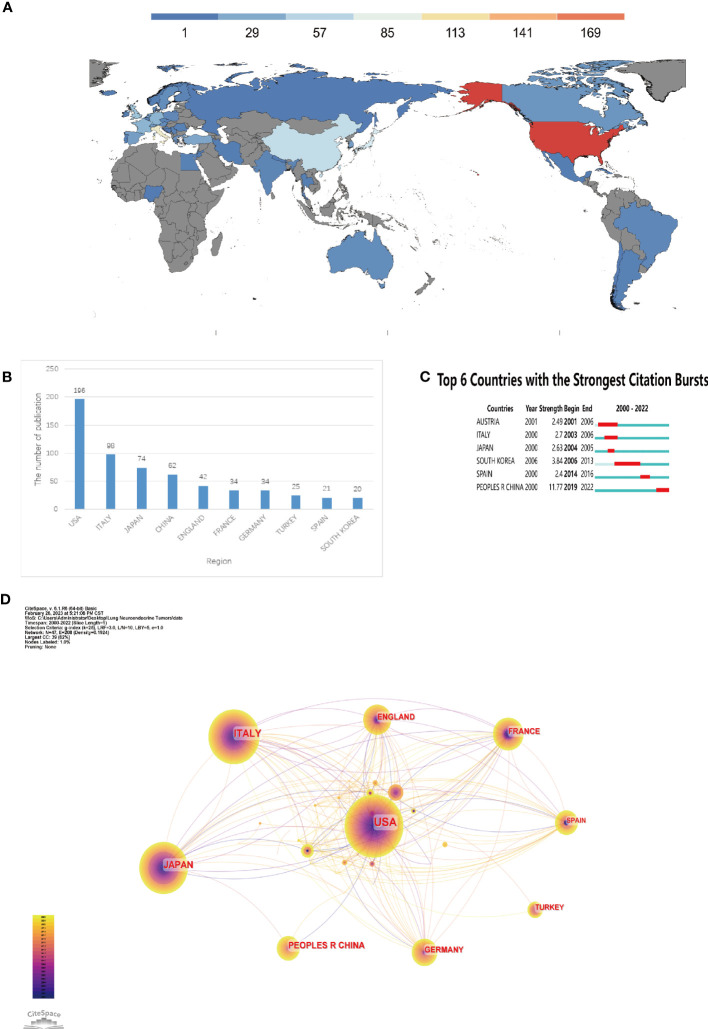
Analysis of countries involved in Lung Neuroendocrine Tumors. **(A)** The distribution of countries by publishing volume. **(B)** The top ten nations in terms of output. **(C)** Countries that have had periods of burst in papers about lung neuroendocrine tumours since 2000. **(D)** country connections appear in a network diagram.

The combined network has 47 nodes and 208 linear connections when we mapped the national and regional distributions (**Figure 2**). The connections between the nodes showed collaborative links, while the size of the nodes denoted the quantity of articles per country. According to the number of articles published (**Figure 2A**) and the bar chart (**Figure 2B**), a total of 594 articles were published over the past 20 years from 47 different nations and regions, with the top 10 countries being composed of 6 European nations (Italy, UK, France, Germany, Turkey, and Spain), 3 Asian nations (Japan, China, and Korea), and 1 North American nation (USA). The top 3 nations and regions were Japan (n = 74, 12.46%), the United States (n = 196, 34.00%), and Italy (n = 98, 16.50%). Despite the fact that France worked closely with more nations and areas than the United States, including the United States, Mexico, Argentina, Chile, and Brazil in the Americas, as well as the United Kingdom, Spain, Russia, and Belgium in Europe, the United States published the most publications overall. China collaborated with fewer nations, primarily Japan, in comparison. According to the burst analysis (**Figure 2C**), research on pulmonary neuroendocrine tumours first gained popularity in Australia, Italy, and Japan, followed by Korea between 2006 and 2013, then Spain, and since 2019 has gradually increased in China, where it has remained popular up to this point (**Figure 2D**).

#### 3.1.3 Institutional analysis

**Figure 3:**
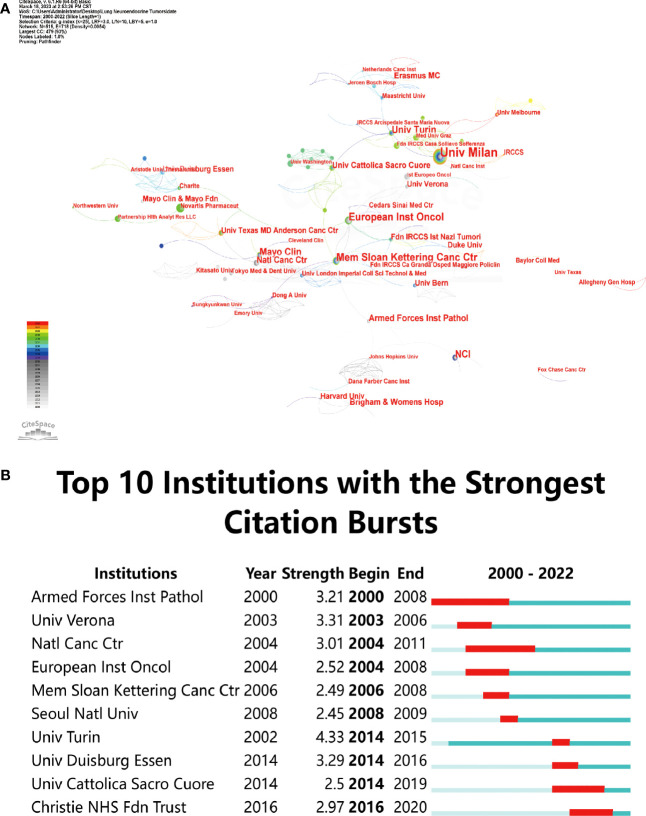
Examination of institutions engaged in research on lung neuroendocrine tumors. **(A)** Network diagram of institutions. **(B)** Institutions in the top 10 burst institutions in articles about lung neuroendocrine tumors that have experienced times of burst since 2000.

A network structure of the combined institutions was created based on an examination of institutional posting volume, as seen in **Figure 3A**. There were 515 nodes and 718 linear connections in the network diagram. Milan University (n = 25, 4.21%), Memorial Sloan Kettering Cancer Center (n = 15, 2.53%), and European Institute of Oncology (n = 15, 2.53%) were the top three organisations. Among the top 10 institutions in terms of number of publications, four institutions Memorial Sloan Kettering Cancer Center, National Cancer Institute, Mayo Clinic College of Medicine, Armed Forces Institute of Pathology were from the United States, and four institutions, Milan University, European Institute of Oncology, Turin University, and Sacred Heart Catholic University, were from Italy, indicating that these two countries made important contributions in the field of neuroendocrine tumors of the lung. The Memorial Sloan Kettering Cancer Center had the highest centrality (centrality=0.21), demonstrating its substantial effect in this area (**Table 1**).

**Table 1:** Ranking of the number of articles published by institutions.

Rank	Count	Centrality	Year	Institutions
1	25	0.08	2003	Milan University
2	15	0.21	2006	Memorial Sloan Kettering Cancer Center
3	15	0.13	2004	European Institute of Oncology
4	12	0.06	2002	Turin University
5	12	0.02	2000	National Cancer Institute
6	11	0.04	2000	Mayo Clinic College of Medicine
7	9	0.09	2000	Armed Forces Institute of Pathology
8	8	0.04	2004	National Cancer Center
9	7	0.05	2014	Sacred Heart Catholic University
10	7	0.01	2010	Erasmus University Medical Center

The burst analysis revealed that famous organisations like the National Cancer Centre, Armed Forces Institute of Pathology, and others had been at the forefront of earlier research on neuroendocrine tumours of the lung. The University of Duisburg-Essen, Turin University, and the European Institute of Oncology have all grown in stature recently. The recent burst of two institutions, one each from Italy and the United Kingdom, demonstrated how popular pulmonary neuroendocrine tumour research has become in these nations lately (**Figure 3B**).

#### 3.1.4 co-cited authors and co-cited journals

There were co-cited 700 authors altogether. TRAVIS WD (n = 288) received the most co-citations, followed by FILOSSO PL (n = 88), MODLIN IM (n = 85), CAPLIN ME (n = 79), and FINK G (n = 75). TRAVIS WD, MODLIN IM, and FINK G are also high centrality authors in the top 5 co-cited authors (**Table 2**), highlighting their remarkable contribution to this field.

**Table 2:** The top 5 Co-cited authors participated in research on Lung Neuroendocrine Tumors.

Rank	Count	Co-cited authors	Centrality	Co-cited authors
1	280	TRAVIS WD	0.14	MODLIN IM
2	88	FILOSSO PL	0.12	PELOSI G
3	85	MODLIN IM	0.11	BRAMBILLA E
4	79	CAPLIN ME	0.1	TRAVIS WD
5	75	FINK G	0.1	FINK G

**Table 3:** The top 5 journals with the most co-citations for work on lung neuroendocrine tumors.

Rank	Count	Co-cited journals	IF	Centrality	Co-cited journals	IF
1	369	American Journal Of Surgical Pathology	6.298	0.08	NATURE	69.504
2	221	Annals Of Thoracic Surgery	5.102	0.08	British Journal Of Cancer	9.075
3	206	CHEST	10.262	0.07	Cancer Research	13.312
4	200	Journal Of Clinical Oncology	50.717	0.07	The New England Journal Of Medicine	176.079
5	190	Journal Of Thoracic Oncology	20.121	0.07	Journal Of Clinical Endocrinology & Metabolism	6.134

As shown in Table 3. American Journal Of Surgical Pathology (n=369) received the most co-citations, followed by Annals Of Thoracic Surgery (n=221), CHEST (n=206), Journal Of Clinical Oncology (n=200), and Journal Of Thoracic Oncology (n=190). It was important to note that the top 5 journals in terms of centrality and quantity of citations all had higher IFs, demonstrating the high calibre of the co-cited journals.

### 3.2 Hot spots and evolution of lung neuroendocrine tumor research based on co-cited literature

#### 3.2.1 Analysis of the number of co-cited literature

**Figure 4:**
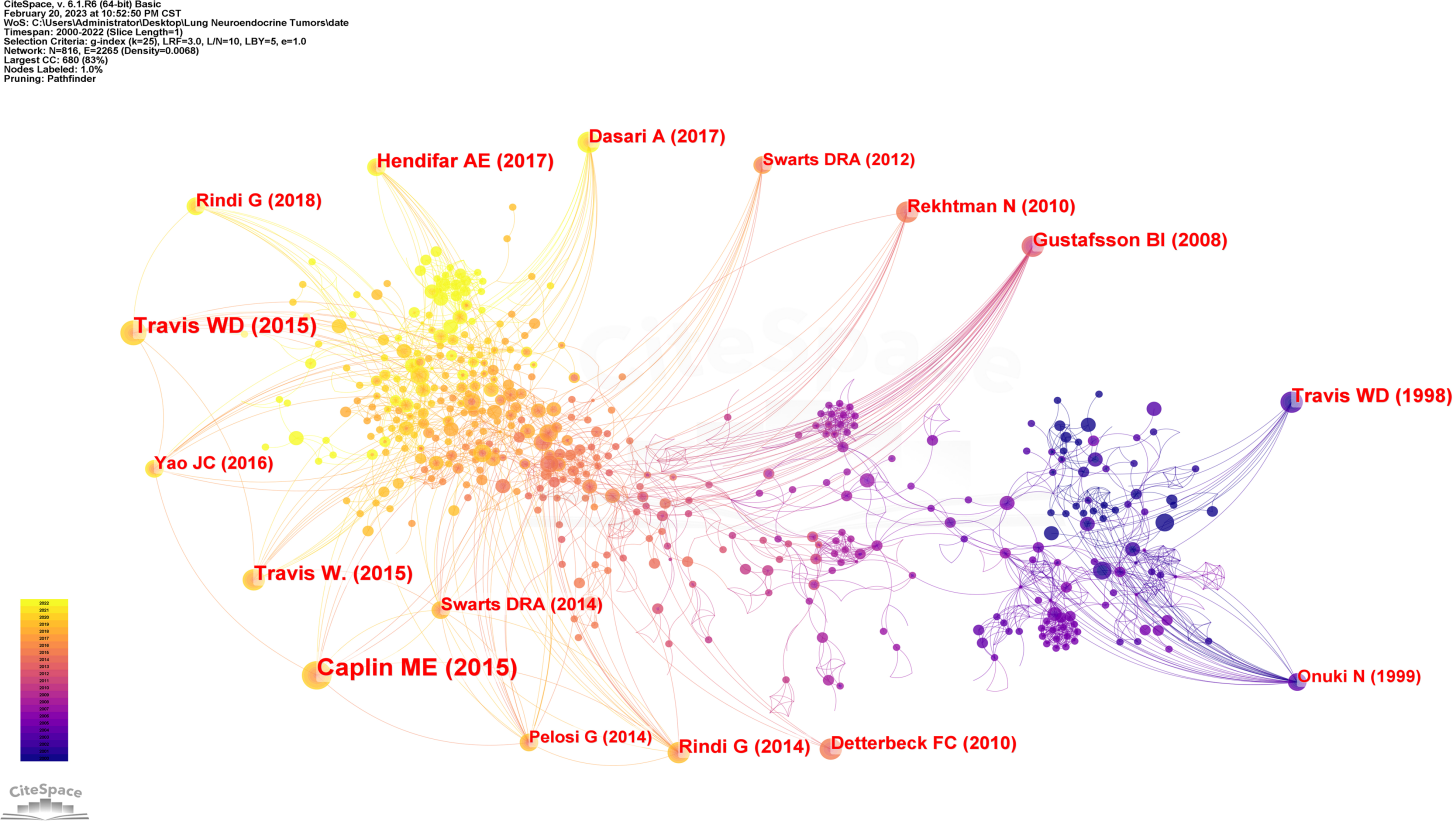
Analysis of cited references involved in Lung Neuroendocrine Tumors.

When two or more pieces of literature were cited by one or more periodicals at the same time, that was what was meant by a co-citation relationship between the two pieces of literature. This connection between nodes in the graph could be used to identify common themes in the literature and serve as a metric for how closely related two articles are (8). Using CiteSpace software, we examined 594 pieces of literature. The network graph of literature co-citations in **Figure 4** included 816 nodes and 2265 links. The co-citations and references of the gathered research were depicted as nodes and lines, accordingly. The number of references to the study increases with node size. The circles in the node’s colour and thickness represented the frequency of references during various time periods. Cooler colours denote earlier years, while warmer colours denote more current years, since the line colours exactly matched the time slices. Yellow or orange lines were used to depict recent co-references. The statistics on the amount of citations were used to determine the literature’s citation ranking (**Table 4**). Clinical research papers made up the majority of the literature, which was arranged according to how frequently references were mentioned. The most highly cited article according to the number of citations was titled Pulmonary neuroendocrine (carcinoid) tumors: European Neuroendocrine Tumor Society expert consensus and recommendations for best practice for typical and atypical pulmonary carcinoids. It was published in 2015 and stated that “pulmonary carcinoid tumours, including typical and atypical carcinoid tumours, are uncommon.” Somatostatin analogues are available for unresectable pulmonary carcinoid tumours, and systemic chemotherapy is available for progressive pulmonary carcinoid tumours, but temozolomide has the greatest clinical benefit. Cytotoxic regimens are of limited effectiveness. The most common cytotoxic regimen is an etoposide and platinum combination (3). In addition, this literature also burst into first strength, suggesting that it is of guiding importance for subsequent research. The second-ranked article in terms of citation count and burst intensity, “The 2015 World Health Organization Classification of Lung Tumors: Impact of Genetic, Clinical and Radiologic Advances Since the 2004 Classification”, reached the following conclusion: “The most crucial histological criterion for differentiating typical carcinoid tumours from atypical carcinoid tumours and carcinoid tumours from high-grade SCLC and LCNEC is careful counting of mitoses.” (9). The key histology criterion for identifying pulmonary neuroendocrine tumours was defined in this article. Notably, William D. Travis authored three of the top ten articles in terms of the number of citations, highlighting his standing as an expert in lung neuroendocrine tumours. Additionally, seven of the top 10 articles were released within the last ten years, showing that peer-reviewed researchers have been paying close attention to the findings regarding neuroendocrine tumours in the lung and that they may continue to be a hot topic in future studies. The 2008 article “ Bronchopulmonary neuroendocrine tumors “ by Gustafsson BI et al., which discusses how improved understanding of lung neuroendocrine tumour biology and tumour genetic characteristics is key to therapeutic evolution, with the main objective being the need to develop early diagnostic tests and establish precisely targeted therapeutic strategies, is also of the utmost importance (4). This article has a centrality of 0.55, which indicates that it significantly influenced later investigations of neuroendocrine tumours in the lung (**Table 4**). Centrality above 0.1 is regarded to be a crucial node.

**Table 4:** Ranking of cited times of cited references.

Rank	Counts	Centrality	Year	Cited references
1	55	0.01	2015	Caplin ME, 2015, ANN ONCOL, V26, P1604, DOI 10.1093/annonc/mdv041 (3)
2	36	0.05	2015	Travis WD, 2015, J THORAC ONCOL, V10, P1243, DOI 10.1097/JTO.0000000000000630 (9)
3	26	0.02	2015	Travis W., 2015, WHO CLASSIFICATION T, V7, P150 (10)
4	23	0.02	2017	Hendifar AE, 2017, J THORAC ONCOL, V12, P425, DOI 10.1016/j.jtho.2016.11.2222 (11)
5	23	0.07	1998	Travis WD, 1998, AM J SURG PATHOL, V22, P934, DOI 10.1097/00000478-199808000-00003 (12)
6	21	0.16	2014	Rindi G, 2014, ENDOCR-RELAT CANCER, V21, P1, DOI 10.1530/ERC-13-0246 (13)
7	21	0.55	2008	Gustafsson BI, 2008, CANCER-AM CANCER SOC, V113, P5, DOI 10.1002/cncr.23542 (4)
8	19	0.05	2017	Dasari A, 2017, JAMA ONCOL, V3, P1335, DOI 10.1001/jamaoncol.2017.0589 (5)
9	17	0.07	2018	Rindi G, 2018, MODERN PATHOL, V31, P1770, DOI 10.1038/s41379-018-0110-y (14)
10	17	0.01	2010	Detterbeck FC, 2010, ANN THORAC SURG, V89, P998, DOI 10.1016/j.athoracsur.2009.07.097 (15)

#### 3.2.2 Analysis on the features of literature co-citation burst

**Figure 5:**
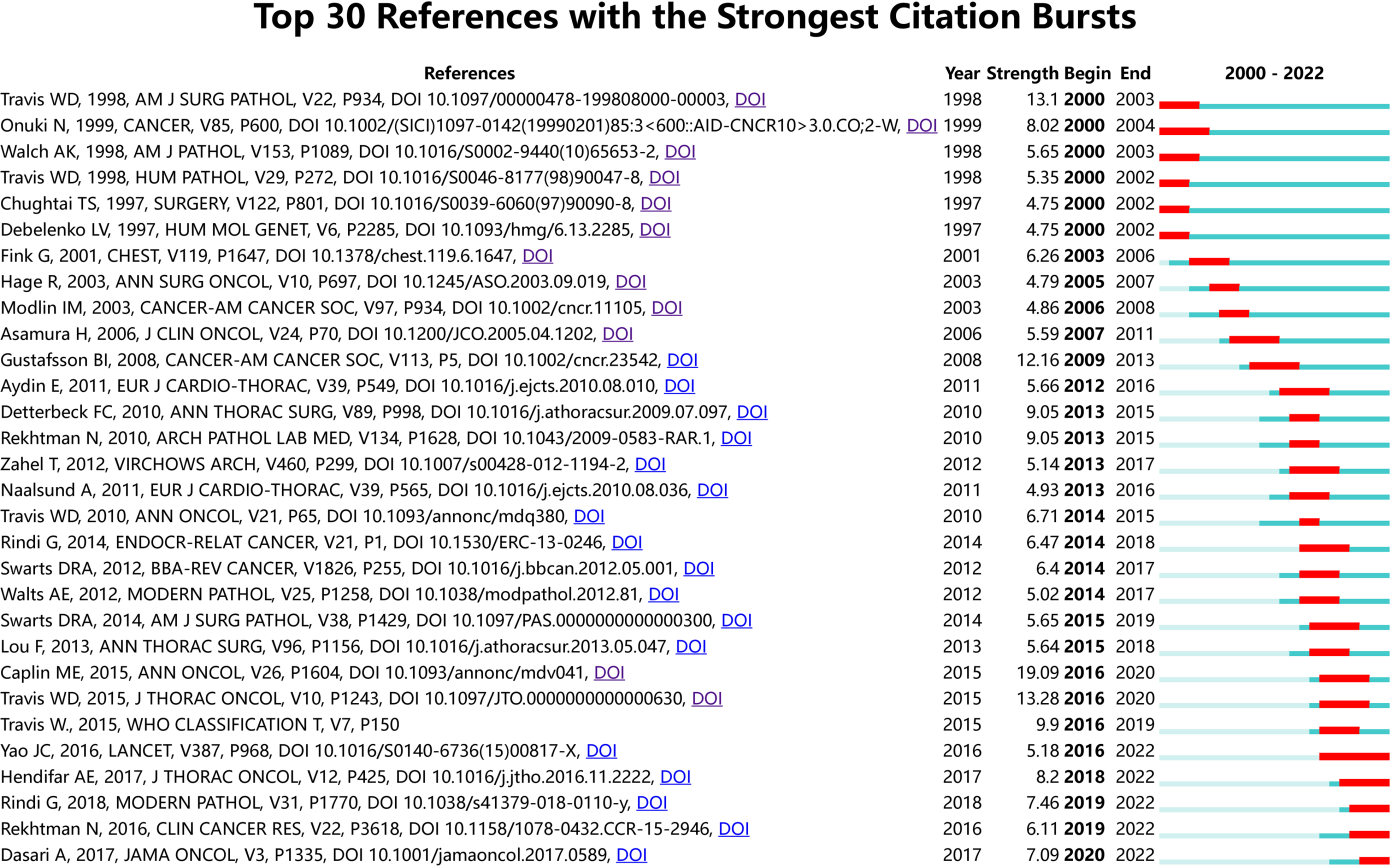
References with periods of burst from 2000 onward among the top 30 burst references in papers about Lung Neuroendocrine Tumors.

The burst strength size was used to gauge how innovative the research findings were; the larger the burst strength, the more innovative the research findings, signifying the field’s frontier (16). Emerging trends in the study of neuroendocrine tumours of the lung were identified by a burst analysis of the co-cited literature, which included works that had drawn the attention of peers (17). The top 30 bursts of literature, as determined by our screening of the gathered literature for burst strength, are depicted in **Figure 5**. The literature ranked first and second in terms of burst strength was repeated above. The third in terms of strength of burst was published in 1998 under the title “Survival analysis of 200 pulmonary neuroendocrine tumors with clarification of criteria for atypical carcinoid and its separation from typical carcinoid”, using a study of 200 lung neuroendocrine tumors, the authors of this article illustrated that mitotic count was the only independent predictor of prognosis in this tumor and that the prognosis of LCNEC and SCLC was similar but both were worse than AC, which in turn was significantly worse than TC (12). The second and third ranked papers in terms of burst strength both pointed to the importance of counting mitoses for lung neuroendocrine tumors, particularly in terms of prognosis and tissue classification.

According to the characteristics of the year of bursting, there were 6 papers bursting from the year 2000, and they described the classification of neuroendocrine tumors of the lung and their prognosis. For example, Chughtai et al. evaluated 84 patients who underwent surgery for bronchial carcinoid tumors and concluded that patients with a histopathological type exhibiting carcinoid tumors and no adverse prognostic factors such as lymph node metastases should be considered for conservative treatment or partial pneumonectomy (18); The MEN1 gene locus is on chromosome 11q13, and loss of heterozygosity (LOH) at this locus has been linked to the aetiology of sporadic pulmonary carcinoid tumours and is a distinctive genetic alteration in this tumour, it was noted (19–21). The following burst of literature focused on the prognostic impact of the histopathological type of lung neuroendocrine tumor (TC, AC, LCNEC or SCLC), with 5-year survival rates decreasing in the following order: TC, AC, LCNEC and SCLC, with no significant prognostic difference between LCNEC and SCLC, with surgery being the best treatment for TC, AC and the preferred surgical approach is parenchymal preservation surgery, with an excellent long-term prognosis for patients with lung carcinoid tumors through early diagnosis and aggressive surgical treatment (4, 22–25). Gustafsson BI believed that in SCLC and LCNEC, radical surgery should be detected and performed as early as possible, but because these tumor types progress rapidly and are highly aggressive and metastatic, metastasis often already present at presentation losing the chance of surgery (4).

Subsequently, in a 2013 paper titled “Phenotyping of pulmonary carcinoids and a Ki-67-based grading approach,” authors Zahel et al. introduced a grading system based on proliferation index (PI) and mitotic count with a view to creating a grading system for pulmonary carcinoids (PC) that distinguishes between PC with low and highly aggressive biological behaviours, a system that is superior to the conventional TC and AC classification methods (26). Travis et al. stated in a 2014 burst of research that: diagnosis of SCLC, TC, and AC can be conducted by light microscopy and in most cases no special tests are necessary, but for LCNEC, NE differentiation needs to be confirmed by immunohistochemistry or electron microscopy (27). A three-tiered grading system based on the Ki-67 index, mitotic count, and necrosis binding generation with specific generation of cut-off values for pulmonary neuroendocrine tumours was mentioned in the literature, which also burst in 2014, to have a valid and reliable prediction (13). The 2014 burst entitled Limited role of Ki-67 proliferative index in predicting overall short-term survival in patients with typical and atypical pulmonary carcinoid tumors, authors Walts et al. noted that the Ki-67 index has a critical value of 5% and can be used as a predictor of prognosis in patients with typical and atypical carcinoid cancers (28). Similarly, in 2015, the literature burst on the conclusion that “classification and prediction of the prognosis of patients with carcinoid tumors according to the Ki-67 proliferation index (<5%; ≥5%) could be improved” by evaluating a randomised group of 123 cases diagnosed with lung carcinoid tumors (29). The authors of the study entitled Pulmonary neuroendocrine (carcinoid) tumors: European Neuroendocrine Tumor Society expert consensus and recommendations for best practice for typical and atypical pulmonary carcinoids burst in 2016, the authors concluded that: somatostatin analoids were first-line therapeutic agents for carcinoid syndromes and could be thought of as first-line systemic antiproliferative therapy for unresectable PC, especially for low-grade TC and AC. For metastatic disease, local or radiation-targeted therapy should be considered, and systemic chemotherapy may be used for progressive PC (3). Bursting with literature in 2016 and 2018, everolimus was the first targeted agent to demonstrate potent antitumor activity and acceptable tolerability in a variety of neuroendocrine tumors, including those originating in the pancreas, lung and gastrointestinal tract, and everolimus was already approved for use in advanced TC or AC in the US. These two publications burst into existence until now, providing novel approaches to treating neuroendocrine tumours of the lung and have a significant and far-reaching impact (11, 30). In conclusion, over the last 20 years, research on neuroendocrine tumours of the lung showed: molecular types were gradually studied in greater depth, and treatments were progressively diversified (surgery, chemotherapy, radiotherapy, targeted therapy).

### 3.3 Exploring the hot spots and evolution of rectal cancer radiotherapy research based on keywords

#### 3.3.1 Analysis of keyword co-occurrence clustering

**Figure 6:**
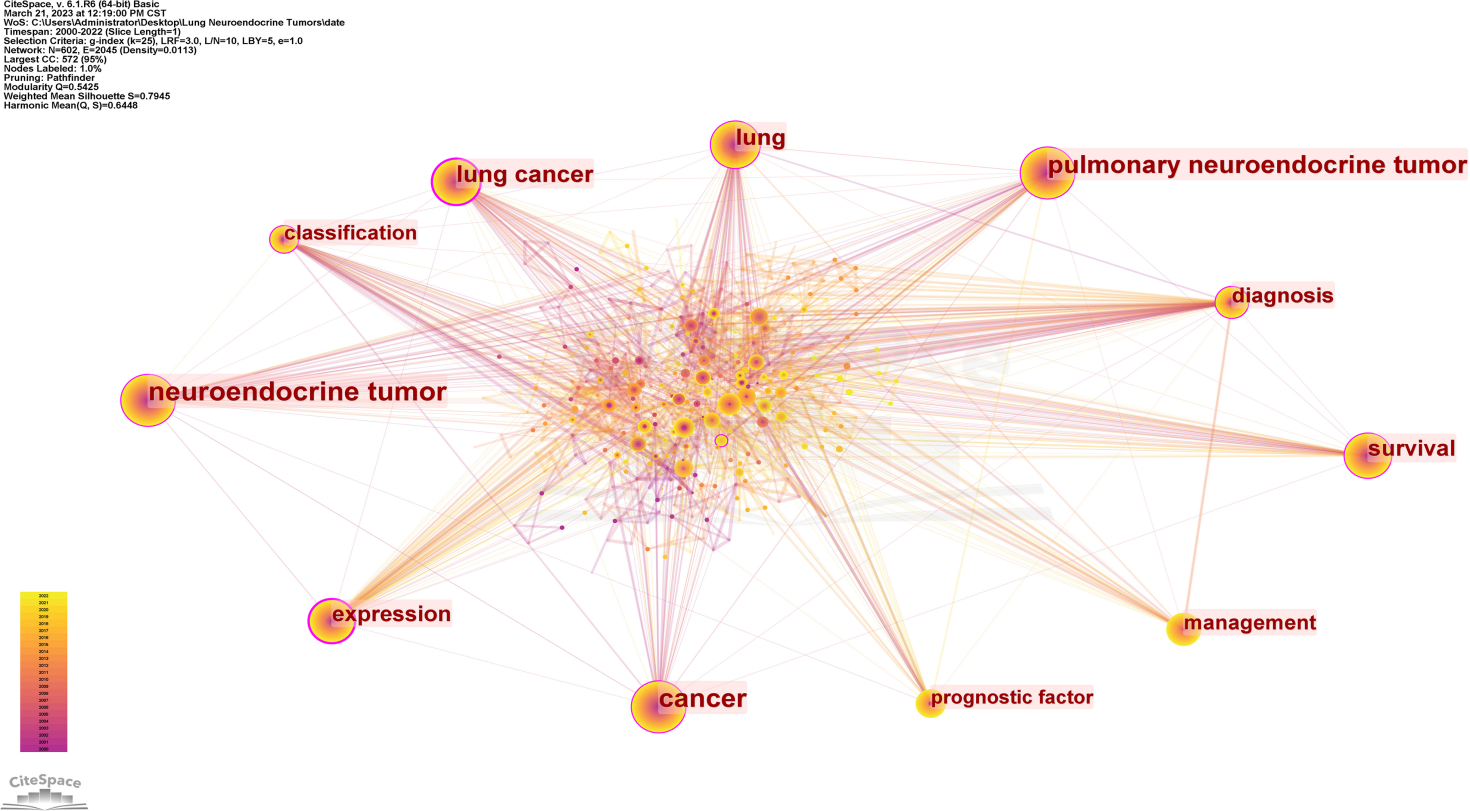
CiteSpace visualization map of keywords analysis.

The keyword was a high-level summary of the article. High-frequency keywords tended to reflect the main research content of a field (31). Keyword co-occurrence analysis was conducted on 594 literatures from 2000-01 to 2022-12, and the time slice was set to 1 year to obtain a keyword co-occurrence map consisting of 602 nodes and 2,045 connections (**Figure 6**). The nodes served as placeholders for keywords, the size of each node reflected the frequency of co-occurrence of the term, and the colours of the nodes and connecting lines represented the order of events as the time moved from far away to close at hand. The top ten keywords that appeared most frequently were: neuroendocrine tumor, cancer, pulmonary neuroendocrine tumor, lung cancer, lung, survival, expression, diagnosis, management, classification. This proved that survival, expression, diagnosis, management, and classification were important parts of lung neuroendocrine tumor research and had a high level of interest. As a hub node, centrality reflected the significance of the node in the network and was usually evaluated with a mediator value ≥ 0.1. Excluding keywords related to the subject term “lung neuroendocrine tumor”, such as “lung cancer”, “lung”, “ neuroendocrine tumor”, “cancer”, etc., the keywords with intermediary values ≥0.1 were shown in **Table 5**, which were the main research hotspots in this field in the past 23 years.

**Table 5:** Ranking of keywords.

Rank	Frequency	Keywords	Centrality	Keywords
1	260	neuroendocrine tumor	0.28	expression
2	231	cancer	0.2	diagnosis
3	176	pulmonary neuroendocrine tumor	0.15	classification
4	123	lung cancer	0.14	chemotherapy
5	106	lung	0.12	survival
6	92	survival		
7	80	expression		
8	73	diagnosis		
9	66	management		
10	57	classification		

The clustering effect of CiteSpace software mapping could be measured by Q and S values, and it was generally accepted that Q > 0.3 would indicate that the delineated clustering structure was significant; S > 0.5 was generally considered to be reasonable, and S > 0.7 indicated that the clustering was efficient and convincing (32). Keyword clustering of keyword co-occurrence profiles yielded **Table 6**, where the top 12 clusters all exceeded 20 in size and had high profile coefficients, with S > 0.7 except for cluster #9, proving that the results we obtained were convincing (**Supplementary Figure 1**). Since the cluster labels couldn’t show all the included keywords, they were summarized according to the research hotspots and directions that each cluster tends to be. The 13 clusters could be summarized under the following themes: mechanism of occurrence (#1, #6), diagnostic tools (#2, #7), ectopic ACTH sign (#0, #11), staging and prognosis (#4, #5, #8, #10, #12), and treatment tools (#3, #9), with the research themes reflecting the current hot topics of research.

**Table 6:** Main clusters of keywords.

Cluster ID	Size	Silhouette	Mean (year)	Label (llr)
0	77	0.768	2008	cushings syndrome; management; cushing syndrome; somatostatin receptor scintigraphy; diagnosis
1	73	0.826	2010	apoptosis; neuroendocrine tumor; phosphorylation; hormone level; neuroendocrine cell
2	62	0.841	2010	adjuvant chemotherapy; positron emission tomography; hypoxia; computed tomography; ct
3	57	0.797	2010	pd-l1; chromosome 10p14-p15; pd-1; loss of heterozygosity; neuroendocrine tumors of the lung
4	51	0.822	2008	typical carcinoid; atypical carcinoid; marker; chromogranin a; atypical carcinoid tumor
5	50	0.709	2009	pulmonary carcinoid tumor; lung neuroendocrine tumors; prognostic factor; quality of life; metastatic disease
6	44	0.727	2007	comparative genomic hybridization; classification; identification; protein; cancer
7	37	0.801	2013	case report; bronchoscopic treatment; aspergillus niger; anti-gamma-aminobutyric-acid b receptor encephalitis; small cell lung cancer (sclc)
8	36	0.848	2009	neuroendocrine tumor; otp; distant metastases; metastasis; beta-catenin
9	28	0.693	2018	sublobar resection); seer; propensity score matching; lobectomy; overall survival
10	26	0.804	2011	small cell carcinoma; nanostring ncounter; large cell neuroendocrine carcinoma; neuroendocrine tumor (net); large-cell neuroendocrine lung cancer
11	25	0.865	2006	acth production; analog; secretion; pituitary; ectopic cusings syndrome
12	6	0.989	2019	pediatric tracheobronchial tumors; pediatric airway tumors; airway team; neuroendocrine tumor; lung

LLR, log-likelihood ratio.

#### 3.3.2 Keyword burst analysis

**Figure 7:**
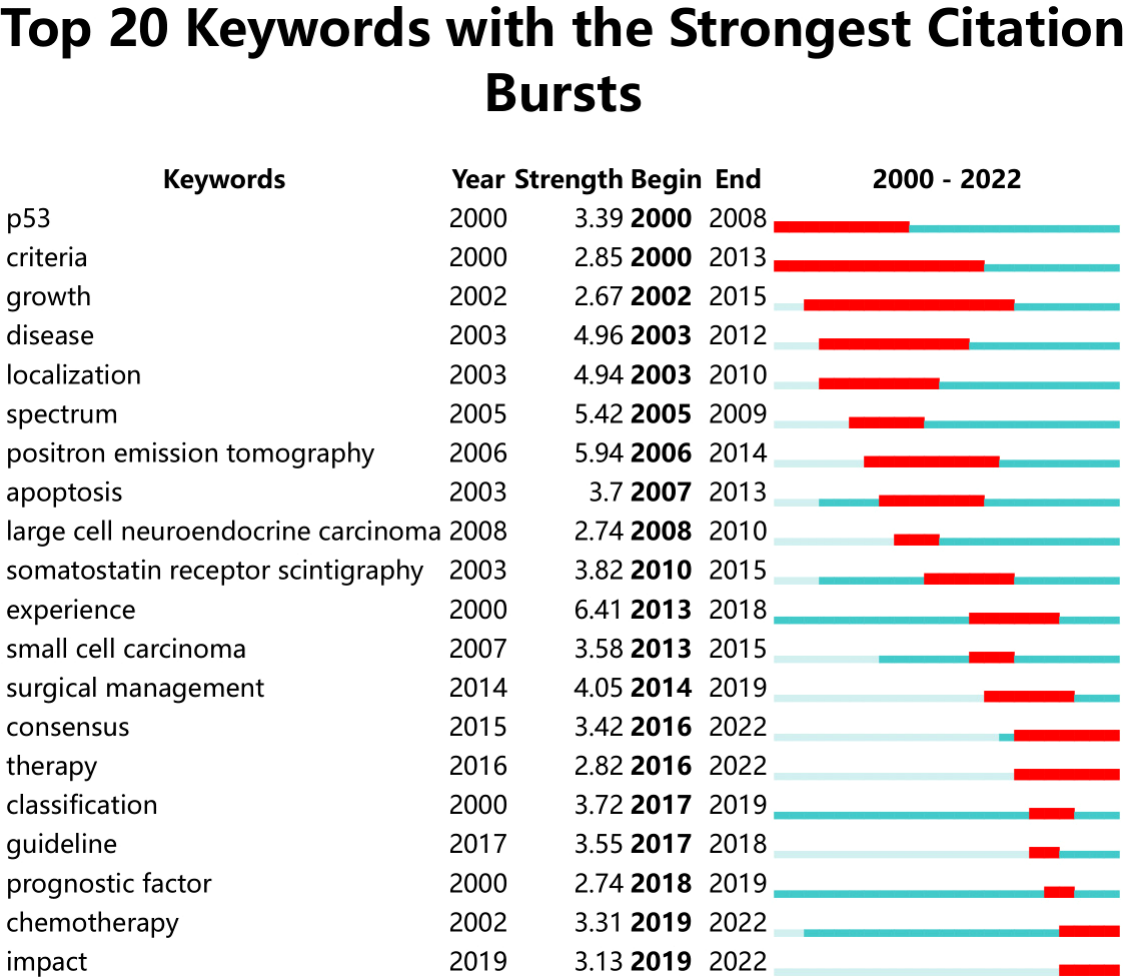
Keywords with periods of burst from 2000 onward among the top 20 burst keywords in publications about Lung Neuroendocrine Tumors.

Keyword burst analysis could capture the trends of the topical research trends in a field, and from this, future research focus could be inferred. As shown in **Figure 7**, the continuing progress in research on neuroendocrine tumors of the lung over the past 23 years was revealed. Among the burst keywords, “experience” had the highest burst strength (Strength= 6.41), which may be related to the fact that most studies in this field are retrospective. The longest-lasting keywords for bursts were “criteria” and “growth”, both of which lasted 14 years and appeared early in the year. In addition, early hotspots of research included the relationship between p53 expression in lung neuroendocrine tumors and the mechanism of this tumorigenesis (33). The keyword “localization” in the 2003 burst indicated that the localization of ectopic ACTH syndrome in lung tumors due to neuroendocrine tumors was a hot topic of research during this period, and the burst keyword “somatostatin receptor scintigraphy” was a method for localizing ectopic ACTH syndrome (34–36). The subsequent burst of “apoptosis, spectrum” suggested that molecular genealogy in this field was a hot topic at the time. The recent burst of “surgical management” in the last few years indicated that surgery is a treatment for TC,AC and is the best method for patients who can achieve R0 resection (37, 38). “therapy”, “chemotherapy” continued to the present day, demonstrating that chemotherapy can benefit patients with neuroendocrine tumours of the lung (3, 39). Treatment tools for neuroendocrine tumours of the lung have been gradually gaining the attention of a wide range of researchers.

## 4 Discussion

In this study, we searched the Web of Science database for literature related to lung neuroendocrine tumours, with the time range set from 2000-01-01 to 2022-12-31. 594 publications from all over the world were obtained after filtering by literature type and language. Visual analysis of country regions, authors, institutions and journals to understand the distribution of interest and research directions in lung neuroendocrine tumor research. The co-occurrence analysis and cluster analysis of the co-cited literature and keywords revealed the research hotspots in the field; the burst analysis of the co-cited literature and keywords reflected the research trends in the field. The co-occurrence analysis, cluster analysis, and burst analysis of the co-cited literature and keywords revealed the areas of research that are very active in the field, respectively. Future researchers may find references and inspiration from the presentation of research hotspots and trends.

### 4.1 Study basic information

In terms of annual publication volume, the overall number of annual publications was on a gradual upward trend, indicating that research related to lung neuroendocrine tumors was receiving more and more attention; in terms of countries and regions, research in this field was mainly concentrated in countries such as the USA, Italy, Japan and China, in addition, there was less collaboration between major research countries. Cooperation and academic exchanges between teams from different regions and institutions should be strengthened to capitalise on the advantages of their respective fields in order to enhance the depth of research in this field. In terms of country bursts, research hotspots in the field were first in Australia, Italy and Japan, followed by increased hotness in South Korea, Spain and finally China, where research was developing rapidly. In terms of institutions, institutions from the United States and Italy accounted for 8 of the top 10 institutional publications, which is consistent with the previous highest number of publications from the United States and Italy; institutions from Italy and the United States also consistently featured in the institutional burst analysis, indicating the important contribution of these two countries in this field. In terms of co-cited authors, TRAVIS WD, MODLIN IM and FINK G 3 had a high number of co-citations and high centrality, showing their outstanding contribution in this field. The American Journal Of Surgical Pathology, Annals Of Thoracic Surgery, CHEST, Journal Of Clinical Oncology and Journal Of Thoracic Oncology were the top 5 co-cited journals. The majority of these co-cited authors had work published in these journals, demonstrating the publications’ strong influence in the field of pulmonary neuroendocrine tumours, and researchers could look for high quality literature in this field based on these authors or journals.

### 4.2 Research hotspot of lung neuroendocrine tumor

#### 4.2.1 Mechanism of occurrence

Abnormal expression of proto-oncogenes and oncogenes, abnormal signaling pathways, and chromosomal variants (gene mutations, gene deletions) may contribute to the development of neuroendocrine tumors in the lung. Loss of heterozygosity (LOH) for material at 11q13 including the MEN1 locus was a distinctive genetic change in this tumor (19–21); Heterozygous deletion (LOH) on chromosome 10p14-p15 associated with lung carcinoid tumor (40); High expression of Sox2 and p63 may affect tumor differentiation in pulmonary neuroendocrine tumors (41); Loss of expression of the OTP gene and the stem cell marker CD44 was associated with poor prognosis (42); fascin immunoreactivity may identify subgroups of lung carcinoid patients with different potential for regional lymph node metastasis (43). Loss of integrity of the E-cadherin/β-catenin complex was strongly associated with lung carcinoid tumorigenesis (44); This provided some basis for subsequent mechanistic studies.

#### 4.2.2 Diagnostic tools

Pulmonary neuroendocrine tumors showed mostly endobronchial nodules or hilar or perihilar masses at CT, with a close anatomical relationship to the bronchi, and calcifications were mostly seen (45); Lung neuroendocrine cancers were diagnosed by F-fluorodeoxyglucose (FDG) PET/CT imaging, which could also be employed for tumour staging (46–48). Bronchoscopic biopsy was also used as the initial diagnostic modality for this neoplasm (49). Somatostatin receptor imaging would be most sensitive to metastatic illness and could detect roughly 80% of initial tumours (3).

#### 4.2.3 Ectopic ACTH sign

Ectopic ACTH syndrome was commonly seen in patients with pulmonary neuroendocrine neoplasms and required radiological and hormonal investigations to detect an extra-pituitary source of ACTH (50, 51). Octreotide scintigraphy may be used as a screening method for ectopic ACTH (36, 52), F-fluorodeoxyglucose (FDG) PET/CT imaging could also be a valid method (53), and somatostatin receptor scintillation imaging could be used for localization during ectopic ACTH sign (34, 54).

#### 4.2.4 Classification and prognosis

The four types of pulmonary neuroendocrine tumours are typical carcinoid tumors (TC), atypical carcinoid tumors (AC), high-grade large cell neuroendocrine carcinoma (LCNEC) and small cell carcinoma (SCLC), and counting mitoses may be a crucial factor in determining the prognosis of pulmonary neuroendocrine tumours (12). Their 5-year survival rates declined in descending order: TC, AC, LCNEC and SCLC, with no discernible difference in prognosis between LCNEC and SCLC (23, 55), and SCLC and LCNEC were rapidly progressive, aggressive and metastatic (4). Also mentioned was the fact that a three-tier grading system based on Ki-67 index, mitotic count and necrosis binding generation, and targeting lung neuroendocrine tumour-specific generation thresholds, yielded data that allowed for a valid prognosis (13).

#### 4.2.5 Treatment tools

Surgery would be the best treatment for TC, AC, with the preferred surgical approach being parenchymal preservation (25), and somatostatin analoges, especially in low-grade TC and AC, may be used as the first-line therapy for unresectable PC. For metastatic disease, local or radiation-targeted therapy should be considered and systemic chemotherapy may be used for progressive PC (3); for patients with SCLC, receiving chemotherapy (platinum plus etoposide is the standard regimen) and radiotherapy may be a better option (56); surgical treatment was performed for resectable LCNEC, and adjuvant chemotherapy was recommended according to SCLC regimen. The targeted therapy “everolimus” was approved in the USA for advanced TC or AC (11).

### 4.3 Research hotspot prediction

The European Society for Neuroendocrinology expert consensus found a gradual decline in survival in pulmonary PC, however, in a US study it was shown that long-term survival in patients with NETs (including pulmonary) has improved, which may be related to improvements in treatment and the discovery of more patients with inert NETs (3, 5). Highly differentiated low-grade NETs (TC and AC) and low-differentiated high-grade NETs (LCNEC and SCLC) showed significantly different survival rates. lCNEC and SCLC showed a higher degree of malignancy, a propensity for early metastasis and a lower 5-year survival rate (24, 57). In recent years, as the molecular mechanisms of pulmonary neuroendocrine tumors have gradually been investigated, more and more therapeutic tools have become available, and “therapy” and “chemotherapy” have burst onto the scene to this day, indicating that therapeutic tools may remain a hot topic of research in the field of lung neuroendocrine tumors for some time to come. However, there was no clear consensus on systemic therapeutic agents applied to pulmonary neuroendocrine tumours other than SCLC (58), and restricting relevant treatments for pulmonary neuroendocrine tumours may reduce their therapeutic efficacy. For example, in two completed phase III studies of advanced pancreatic NET and advanced extrapancreatic NETs in China, surufatinib showed favourable results, and the therapeutic efficacy of surufatinib in evaluating primary neuroendocrine tumours of the lungs is currently being investigated in relation to the novel mechanism of surufatinib to act for a growing group of patients with an unmet need for healthcare (59). Moreover, the current clinical studies suffered from small sample size and lack of randomised clinical trials, so it was suggested that the sample size should be expanded and more scientifically rigorous randomised controlled trials should be used for clinical studies in the next clinical observations.

To the best of our knowledge, this study was the first bibliometric analysis to concentrate on the research of lung neuroendocrine tumours. This article obtained the relevant literature in this field from the WoSCC database and analysed it using CiteSpace software. The data analysis was fairly thorough and objective, and it may highlight the most active areas of study for lung neuroendocrine tumours. Inevitably, of course, there were some limitations to this study. Firstly, we downloaded English language literature from the WoSCC database for the time period from 2000-01-01 to 2022-12-31, and literature from other timeframes and languages were excluded, yielding results that may not be comprehensive. According to the calculation method of CiteSpace software and in combination with our screening criteria, high-quality articles published in recent years were less likely to be included in the study because they were cited less frequently, which may result in a possible underestimation of papers on new therapeutic approaches. In the future, articles from more databases and in more languages need to be included in studies. The recently published literature also needed to be tracked to facilitate peer researchers. In fact, the literature indexed in the Web of Science database was thought to be of high quality and relatively comprehensive, and we still believed that our study covered the vast majority of articles in the field of lung neuroendocrine tumor research and could be used to describe research trends in the field from 2000 to 2022.

## 5 Conclusion

This study was based on a bibliometric approach, applying CiteSpace to visualise and analyse the literature on pulmonary neuroendocrine tumor research in terms of annual number of articles, authors, institutions and keywords. The findings demonstrated that over the previous 20 years, research in this area has advanced significantly. As research progressed, the focus of research in this discipline altered somewhat as it developed, with research on diagnostic tools, mechanisms of occurrence, ectopic ACTH signs, staging and prognosis, and treatment being the main focus of research. Updates and options for treatment may be a research trend in the field of lung neuroendocrine tumors for some time to come. This may provide new approaches to the future treatment of neuroendocrine tumors in the lung, while promoting further development in the field.

## Data availability statement

The original contributions presented in the study are included in the article/**Supplementary Material**. Further inquiries can be directed to the corresponding author.

## Author contributions

SH, MG and YX are co-first authors of this paper, contributed to all tables and figures and the main manuscript. MG and YX downloaded and collated the relevant papers. XZ conceived of the study and provided the methodological guidance. ZC, ZH, YL, XA and GZ revised and supplemented the manuscript. All authors contributed to the article and approved latest version.

## Supplementary material

Data Sheet 1 Strategies for literature search in pulmonary neuroendocrine tumor.
