# Supplementary material for: Visual analysis of lung neuroendocrine tumors based on CiteSpace knowledge graph
Source: Front Endocrinol (Lausanne). 2023 Sep 6;14:1214404. doi: 10.3389/fendo.2023.1214404 (PMC10516576; doi:10.3389/fendo.2023.1214404)
Supplement: Data Sheet 1 — Strategies for literature search in pulmonary neuroendocrine tumor. [file DataSheet_1.docx]

**Strategies for literature search in lung neuroendocrine tumors：**

Databases: SCI-EXPANDED, CPCI-S, CPCI-SSH, BKCI-S, BKCI-SSH

#1: TI= ("bronchial neuroendocrine tumor*") OR TI= ("bronchial carcinoid tumor*") OR TI= ("pulmonary carcinoid") OR TI= ("pulmonary typical carcinoid") OR TI= ("pulmonary atypical carcinoid") OR TI= ("Pulmonary neuroendocrine tumor*") OR TI= ("lung neuroendocrine tumor*") OR TI= ("neuroendocrine tumor* of the lung")

#2: AB= ("bronchial neuroendocrine tumor*") OR AB= ("bronchial carcinoid tumor*") OR AB= ("pulmonary carcinoid") OR AB= ("pulmonary typical carcinoid") OR AB= ("pulmonary atypical carcinoid") OR AB= ("Pulmonary neuroendocrine tumor*") OR AB= ("lung neuroendocrine tumor*") OR AB= ("neuroendocrine tumor* of the lung")

((#1 OR #2) AND DT=(Article)) AND LA=(English)

DOP: 2000-01-01 to 2022-12-31


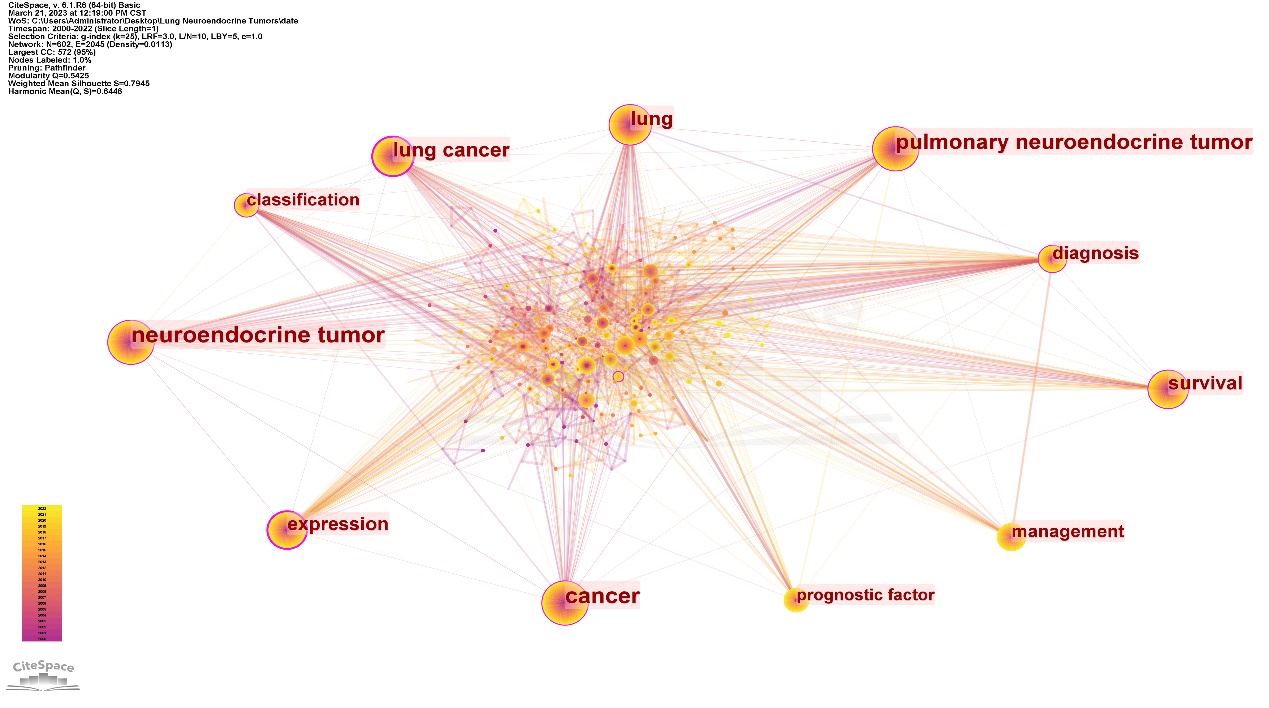


Figure 1 CiteSpace visualization map of keywords analysis.
